# Passively Q-switching an all polarization-maintaining erbium-doped fiber laser with a rhenium disulfide (ReS_2_) saturable absorber

**DOI:** 10.1016/j.heliyon.2023.e20678

**Published:** 2023-10-05

**Authors:** Mahmoud Muhanad Fadhel, Abdulwahhab Essa Hamzah, Norazreen Abd Aziz, Mohd Saiful Dzulkefly Zan, Norhana Arsad

**Affiliations:** Department of Electrical, Electronic and Systems Engineering, Faculty of Engineering and Built Environment, Universiti Kebangsaan Malaysia (UKM), Bangi, 43600, Selangor, Malaysia

**Keywords:** Fiber laser, Polarization maintaining, Q-switching, Rhenium disulfide, Saturable absorber

## Abstract

This study demonstrates a linearly polarized Er-doped fiber laser system featuring an all-polarization-maintaining (all-PM) architecture. Short pulses were generated by Q-switching operation based on drop-casting rhenium disulfide (ReS_2_) saturable absorber (SA) onto a fiber connector placed inside the laser cavity. The Q-switching operation of the laser was able to self-start at a low (23 mW) threshold power of the pump and without the need to use a polarization controller. The proposed laser was able to produce stable pulses with a center wavelength and 3-dB bandwidth of 1558.4 nm and 0.13 nm, respectively. The shortest pulse duration measured (2.8 μs) was achieved at a repetition rate of 37.6 kHz while the highest average output power and pulse energy were 2.2 mW and 76.5 nJ, respectively. Furthermore, as the cavity of the laser was designed to be all-PM the laser that it produced was linearly polarized and had a degree of polarization (DOP) at the level of 94.5 % and 40 dB polarization extinction ratio (PER). Therefore, the proposed laser is a suitable light source for optical applications in environments that are complex.

## 1 Introduction

A Q-switched fiber laser that can perform Q-switching using a saturable absorber (SA) is a highly efficient and compact laser that can generate short and intense pulses of light would be ideal for multiple scientific applications; such as laser processing [1], laser-based medical treatments [2], distributed optical fiber sensing [3], and to produce frequency combs [4,5]. The Q-switching operation can be produced in a laser by inserting a saturable absorber (SA) material into its cavity [6,7]. Several SA materials have been used to effectively produce laser pulses that are Q-switched [[8], [9], [10], [11]]. Among these materials, transition metal dichalcogenides (TMDs) are relatively easy to exfoliate and possess high nonlinear optical response [12]. While the well-known TMDs from group VI (such as MoS_2_, WS_2_, MoSe_2_, WSe_2_, and others) remain popular, a noteworthy TMD called ReS_2_, belonging to group VII, has recently garnered substantial attention. This interest stems from its unique distorted 1T structure with weak interlayer coupling [13,14]. Unlike group VI TMDs, ReS_2_ maintains a direct bandgap across both monolayer and bulk forms as a result of the distorted 1T structure, preventing any significant changes to its optical, chemical, and electrical properties [15,16]. ReS_2_ is a good candidate as a saturable absorber material for pulsed lasers because of its large modulation depth, broad absorption bandwidth, high damage threshold, and relatively low-cost [17,18].

Multiple extant studies have successfully employed ReS_2_ SA in the cavity of fiber lasers to produce Q-switched laser pulses in different wavelength ranges. For instance, Lu et al. (2019) used ytterbium-doped fiber as a gain medium to produce Q-switched laser beams that had a 1047 nm wavelength, 1.56 μs pulse duration, and 3.2 mW average output power [17]. Meanwhile, Mao et al. (2018) produced high energy Q-switched pulses that reached 62.8 μJ in the 1.5 μm range [19]. He et al. (2021) used low-cost rhenium and sulfur powder to prepare ReS_2_ nanosheets instead of using expensive ReS_2_ single crystals as raw material. They used the liquid phase exfoliation method, which is a relatively simple and inexpensive process. The prepared ReS_2_ nanosheets were utilized as a saturable absorber within an Er-doped Q-switched fiber laser, resulting in a minimum pulse duration of 2.4 μs and an output power of 1.25 mW [20].

A fiber laser that is capable of producing outputs that are linearly polarized would be considered a high-performance Q-switching laser as it would have higher polarization stability. This, however, requires the use of a polarization-maintaining (PM) fiber to accomplish. Laser cavity that only contains standard non-PM fibers are incapable of starting the pulsed laser operation on their own and require a polarization controller. This not only leaves them susceptible to environmental perturbations, such as temperature fluctuations and movement of the fibers; but leaves the output polarization state undefined. PM Q-switched fiber lasers have been demonstrated using various nanomaterials. In 2012, Sobon et al. demonstrated the generation of a linearly polarized Q-switched pulses using reduced graphene oxide (rGO) as a saturable absorber. The laser could generate 1.85 μs pulses with 125 nJ energy at 115 kHz repetition frequency [21]. In 2016, Bogusławski et al. deposited antimony telluride (Sb_2_Te_3_) on the surface of a side-polished fiber. This allowed them to exploit the evanescent field interaction between the laser beam and the Sb_2_Te_3_, which resulted in a much higher pulse energy up to 152 nJ and a maximum recorded output power of 18.06 mW [22]. Guo et al. (2016) investigated the stability of tungsten diselenide (WSe_2_) as a saturable absorber material in an all-PM Q-switched erbium-doped fiber laser. They observed stable Q-switched operation for 1.6 h under a pump power of 280 mW [23]. Chen et al. (2023) reported a single-frequency linearly polarized Q-switching operation at 1.06 μm based on Nb_2_GeTe_4_ SA. Stable pulses with the shortest pulse width of 1.36 μs, a linewidth of 28.4 MHz, and a polarization extinction ratio of about 30 dB were observed for 7 h under the pump power of 340 mW [24]. However, the feasibility of using ReS_2_ SA in PM Q-switched fiber lasers have not been investigated yet.

Therefore, this present study proposes placing ReS_2_ SA in the all-PM Er-doped fiber laser cavity for passive Q-switching. The cavity of the laser solely contained fibers and components that were all-PM to ensure that it produced a linearly polarized laser beam output. It was determined that the laser's degree of polarization (DOP) was 94.5 % while its polarization extinction ratio (PER) was 40 dB. The ReS_2_ SA was synthesized via the liquid-phase exfoliation (LPE) method and deposited on the fiber connector by drop casting technique. A low threshold pump power (23 mW) was used to produce the Q-switched pulses without a polarization controller. The stable pulses obtained at 1558.4 nm had a pulse duration of 2.8 μs and repetition rate of 37.6 kHz while the average output power and pulse energy were 2.2 mW and 76.5 nJ, respectively. Therefore, the proposed laser was not only capable of self-starting but produced beams that were linearly polarized. As such, it is a high-performance light source with potential fiber optic sensor and spectroscopy applications.

## 2 Preparation and optical characteristics of the saturable absorber (SA)

### 2.1 Preparing the ReS_2_ SA

**Fig. 1:**
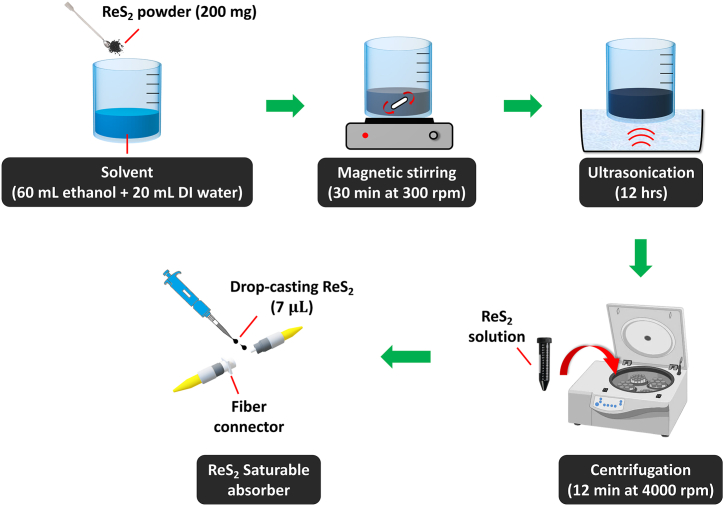
The preparation process of the ReS_2_ SA.

As LPE is a simple and cost-effective method of producing high quality two-dimensional (2D) nanostructures, it was used to prepare the ReS_2_ SA [25,26]. Fig. 1 provides a comprehensive overview of the process that was used to prepare the ReS_2_ solution. More specifically, 200 mg of ReS_2_ powder from Nanjing Muke Nano Technology Co., Ltd. Was first added to a solvent of 60 ml ethanol and 20 ml deionized water. The concoction was then stirred at room temperature in a magnetic stirrer for 30 min at 300 revolutions per minute (rpm). The resulting solution contained 2.5 mg of ReS_2_ per milliliter. After 12 h of ultrasonication, the now homogenous mixture was centrifuged for 12 min at 4000 rpm to eliminate large sediments. The resulting supernatant was then collected and stored. The ReS_2_ SA was then fabricated via drop-casting. More specifically, a micropipette was used to deposit 7 μL of the prepared ReS_2_ solution onto a fiber connector. It was then left to dry for an hour at room temperature before an optical adapter was used to link it to another clean fiber connector.

### 2.2 Characterizing the ReS_2_ solution

**Fig. 2:**
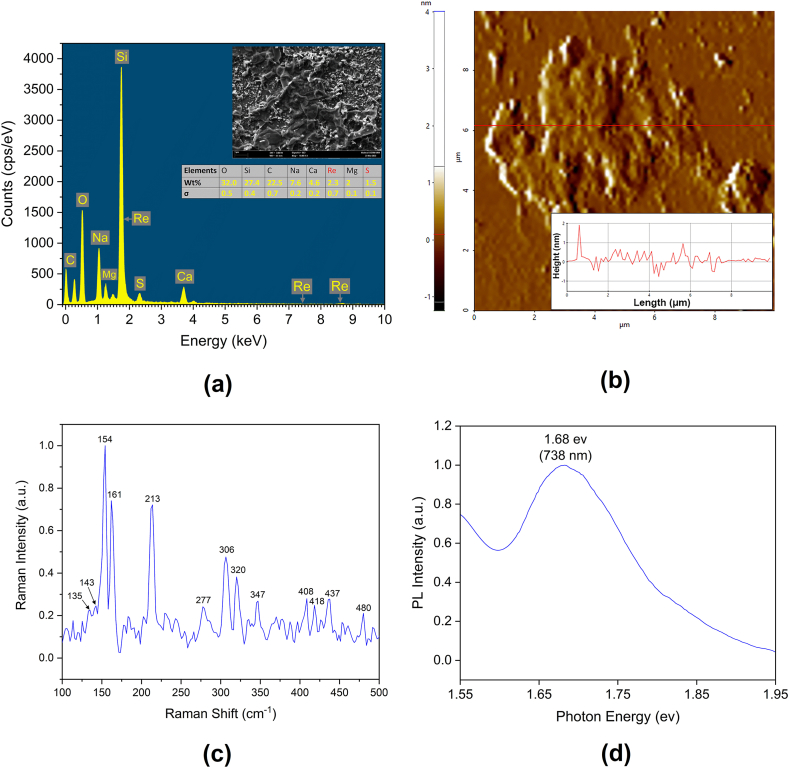
(a) EDX measurement of the prepared sample indicating traces of Re and S atoms. Inset: FESEM image. (b) AFM image of the ReS_2_ sample with 10 × 10 μm area. Inset: the corresponding height profile. (c) Raman spectra of the ReS_2_ sample. (d) Photoluminescence spectra of the ReS_2_ sample.

Drops of the ReS_2_ solution were first deposited on separate glass slides before they were placed in a desiccator to dry. Prior to testing, the dried ReS_2_ samples were coated with iridium (Ir) to ensure that high quality images were produced. A field emission scanning electron microscope (FESEM) and an energy dispersive X-ray (EDX) spectroscope were then used to identify the elemental composition and characterize the surface morphology of the prepared sample. As seen in Fig. 2a, the EDX elemental mapping results prove the presence of rhenium (Re) and sulfur (S). The highest peak observed at ∼1.75 keV was the silicon (Si) of the glass slide onto which the ReS_2_ solution was deposited. The ReS_2_ solution was not applied uniformly to the glass slide, leaving some areas where it was not present. The elements Na, Mg, and Ca appeared on the glass slide as impurities. The inset seen in Fig. 2a is an FESEM image of the ReS_2_ sample at a magnification of 10.0 k. As shown in Fig. 2b, the atomic-force microscope (AFM) image was used to identify the thickness of the prepared ReS_2_ sample. The inset of Fig. 2b shows the corresponding height profile where the thickness was determined to be ∼2 nm which corresponds to ∼3 layers on the basis of the monolayer ReS_2_ of 0.7 nm [27]. Meanwhile, as seen in Fig. 2c, the multiple peaks observed between 100 and 500 ${\text{cm}}^{-1}$ in the Raman spectroscopy results could be attributable to the Ag and Eg Raman modes [28]. Photoluminescence (PL) spectroscopy was utilized to identify the bandgap of the material, as seen in Fig. 2d. The measurements were conducted at room temperature using Edinburgh Instrument FLS920 spectrometer. A 450 W Xenon lamp was used as the excitation source, with a wavelength of 300 nm. The PL spectrum reveals a peak in intensity at ∼1.6 eV (738 nm), which is consistent with the findings of extant studies [29,30].

### 2.3 Determining the optical properties of the ReS_2_ SA

**Fig. 3:**
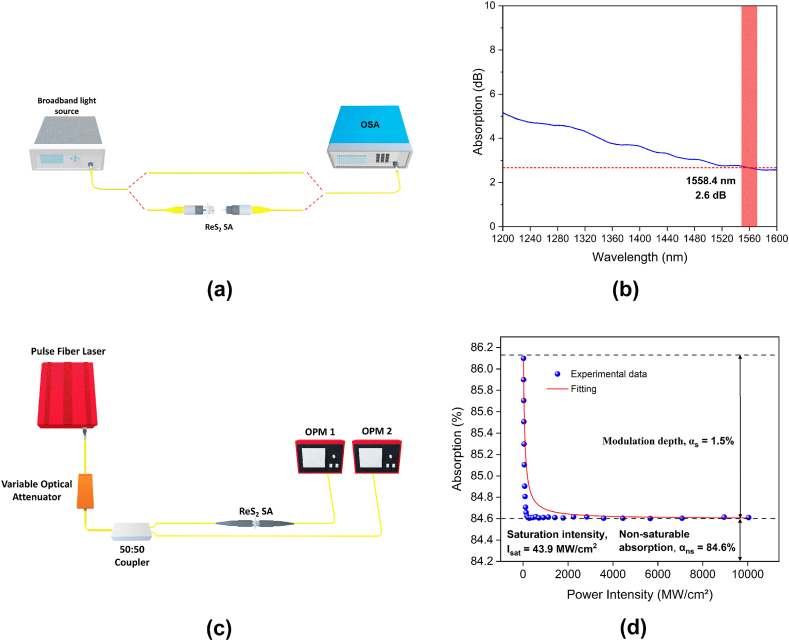
Linear absorption measurement (a) experimental setup and (b) absorption profile of ReS_2_ SA; nonlinear absorption measurement (c) experimental setup and (d) nonlinear absorption curve of ReS_2_ SA.

As seen in Fig. 3a, to determine the linear optical absorption of the ReS_2_ SA between 1200 and 1600 nm, a broadband amplified spontaneous emission (ASE) light source was transmitted through it. A Yokogawa AQ6370D optical spectrum analyzer (OSA) was then used to measure the output signal at 0.02 nm resolution. The ASE light was first connected directly to the OSA to obtain a reference spectrum before the ReS_2_ SA was inserted. As seen in Fig. 3b, which plots the absorption profile of the ReS_2_ SA, the absorption loss was approximately 2.6 dB at 1558.4 nm. The nonlinear optical absorption of the ReS_2_ SA was then determined using a balanced twin-detector setup as shown in Fig. 3c. The pulse source was a homemade mode-locked fiber laser operating at a 1550 nm wavelength, 4.14 ps pulse duration, and 1 MHz repetition rate with a variable optical attenuator (VOA) to adjust the power. An optical coupler (OC) was used to split the power equally and direct it to two separate optical power meters for data collection; namely OPM1 and OPM2. The ReS_2_ SA was attached to the port that connected to OPM2. Therefore, OPM1 was maintained as the reference power. The key parameters that determine the nonlinear saturable absorption of the SA are the modulation depth ($\alpha_{s}$), saturation intensity (${Ι}_{sat}$), and non-saturable absorption ($\alpha_{ns}$), as described in Eq. (1) below [31,32];

(1) $$\alpha \left(Ι\right)=\frac{\alpha_{s}}{1+\frac{Ι}{{Ι}_{sat}}}+\alpha_{ns}$$

where, α is the coefficient of absorption, and Ι is the intensity of the incident light, $\alpha_{s}$ is measured by determining the difference between the linear absorption coefficient at low input intensities and the saturated absorption coefficient at high input intensities, ${Ι}_{sat}$ is the intensity required to decrease the absorption by 0.5 $\alpha_{s}$, and $\alpha_{ns}$ is usually unwanted losses that cannot be saturated by the incident light. Fig. 3d shows the level of absorption at multiple input intensities, where the dots represent the measured data and the curve line corresponds to the fit using Eq. (1). The modulation depth, saturation intensity, and the non-saturable absorption were measured to be 1.5 %, 43.9 MW/cm^2^ and 84.6 %, respectively.

## 3 Pulsed fiber laser application

**Fig. 4:**
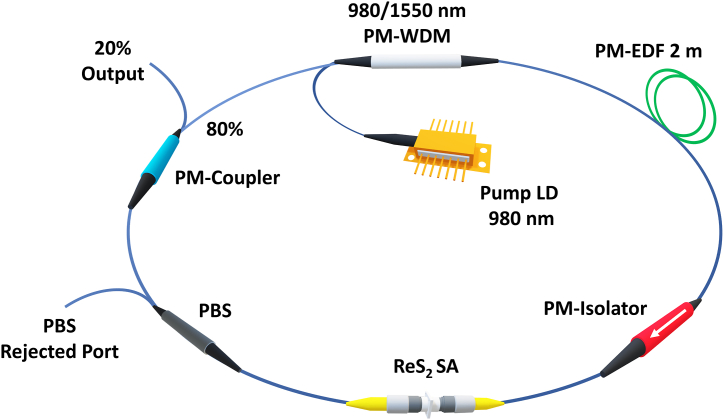
Experimental setup of the all-PM Erbium-doped fiber laser cavity Q-switched using ReS_2_ SA.

The experimental setup of the proposed laser was depicted in Fig. 4. A 2-m-long PM fiber that had been doped with Er ions was placed in the cavity of the laser to serve as the amplifier medium. The 980 nm laser diode through the 980/1550 nm PM wavelength-division multiplexer (PM-WDM) was then used to excite the gain medium. A PM-isolator was used to ensure unidirectional operation and decrease laser reflections while a Thorlabs PBC1550SM-FC polarization beam splitter (PBS) was placed inside the cavity to ensure that the laser only propagated in one polarization axis. An 80/20 p.m.-coupler was attached to monitor the performance of the laser through the 20 % port.

A Yokogawa AQ6370D OSA was used to determine the spectral properties at a resolution of 0.02 nm. A Keysight DSO7104B 1 GHz-oscilloscope, a Keysight N9320A 3-GHz radio frequency spectrum analyzer, and a Keysight 15-GHz 11982A photodetector were used to determine the temporal properties. A Thorlabs PM100D optical power meter was used in conjunction with a Thorlabs S122C photodiode power sensor operating between 700 and 1800 nm to determine the average output power of the laser.

Inserting the ReS_2_ SA into the cavity of the laser facilitated the generation of stable pulses that were Q-switched when the power of the pump was 23 mW. However, when the power of the pump exceeded 67 mW, the pulse train collapsed and switched the laser to continuous-wave (CW) operation. Nevertheless, the Q-switched pulses were easily restored by decreasing the power of the pump to below 67 mW. Instead of thermal damage, this phenomenon was more likely caused by ReS_2_ SA oversaturation [33].

**Fig. 5:**
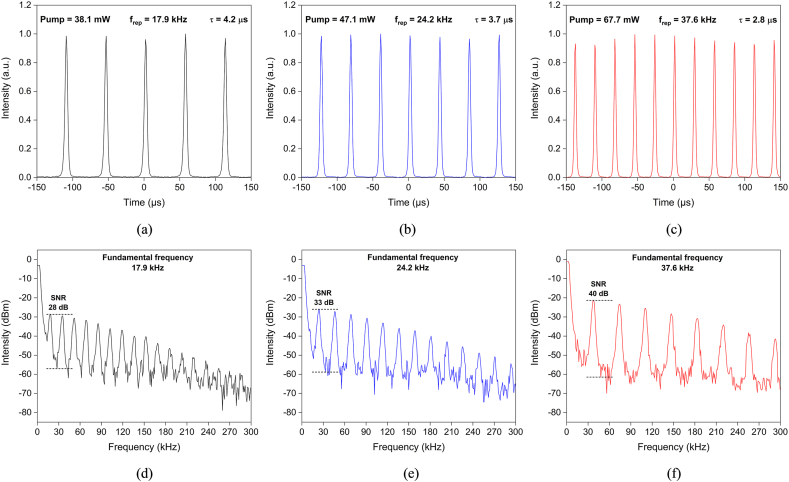
(a–c) Oscilloscope trace of Q-switched pulses and (d–f) the corresponding RF spectrum at different pumping powers.

Fig. 5a-c depicts the oscilloscope trace of the pulses that had been Q-switched and their related radio frequency (RF) spectrums in Fig. 5d-f at varying pumping powers. The fundamental frequency of the RF spectrum was found to match well with the frequency recorded by the oscilloscope. Furthermore, the RF spectrum's signal to noise ratio (SNR) was 40 dB when the pumping power was at maximum.

**Fig. 6:**
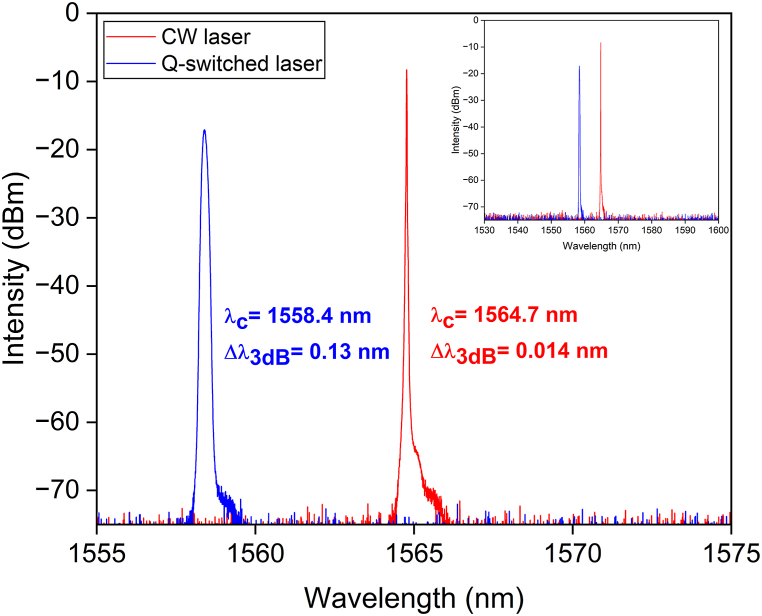
Output spectrum of CW laser (red) and Q-switched laser (blue).

**Fig. 7:**
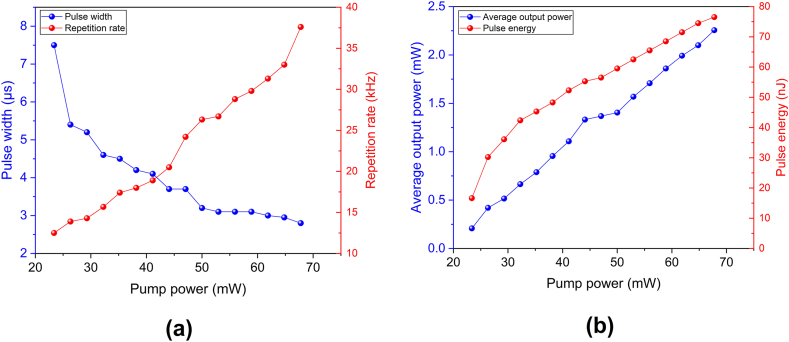
Evolution of Q-switched pulses performance as a function of pump power: (a) pulse width and repetition rate, (b) average output power and pulse energy.

The output spectrum of the Q-switched pulses and the CW laser is shown in Fig. 6. When the ReS_2_ SA was inserted in the cavity of the laser, the peak wavelength and the 3 dB spectral bandwidth of the Q-switched output shifted from 1564.7 to 1558.4 nm and from 0.014 to 0.13 nm, respectively. This center wavelength shifting occurs due to the higher insertion losses inside the cavity with the employment of the SA. The laser starts to lase at a shorter wavelength, which has a higher gain to compensate for the losses [34]. Fig. 7a depicts the Q-switched laser's repetition rates and pulse widths at pump power ranges of 23–67 mW. When the pump power increased, the repetition rate increased from 12.5 to 37.6 kHz while the pulse width decreased from 7.5 to 2.8 μs. Fig. 7b depicts the correlation between the average output power and pulse energy as a function of pump power. When the power of the pump was at its highest (67 mw), the highest average output power was 2.2 mW while the highest pulse energy was 76.5 nJ.

**Fig. 8:**
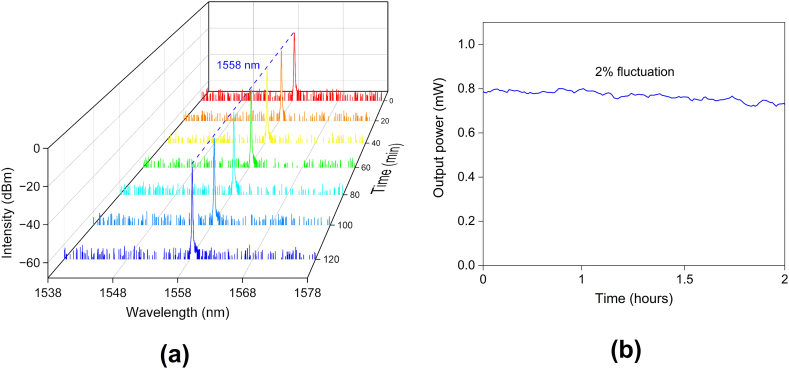
The operating stability of the Q-switched fiber laser over a 2 h span. (a) Spectrum; (b) output power fluctuations with 2 % standard deviation.

The stability of the proposed Q-switched laser has been expressed in Fig. 8. Under continuous pumping at ∼35 mW, it can be observed that there is no significant drift in the central wavelength of the spectrum over the measuring period of 2 h, as depicted in Fig. 8a. A Thorlabs PM100D optical power meter was used to measure the output power to determine the stability of the all-PM fiber ring cavity. Fig. 8b depicts the results with 2 % standard deviation for fluctuations.

**Fig. 9:**
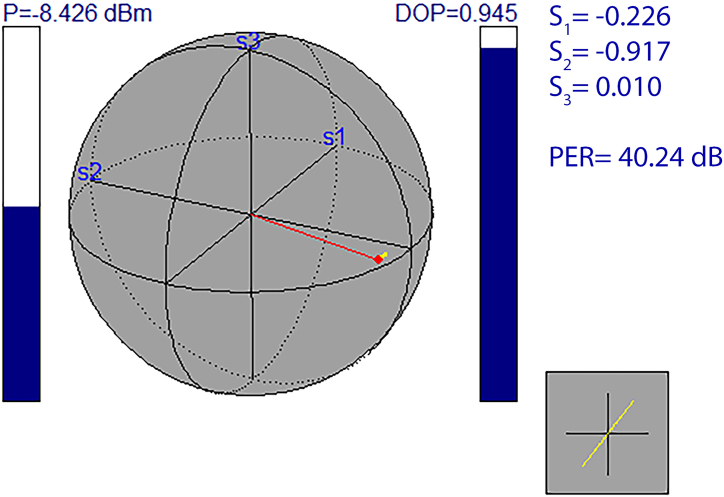
Polarization parameters of the Q-switched fiber laser.

All the fibers and components used in the resonator were PM and spliced with a Fujikura FSM-100P specialty fiber fusion splicer to match the proper polarization axis and ensure the output of the laser was linearly polarized. A polarization beam splitter was also used to split the single input into two linear polarization outputs. A Keysight N7781B polarimeter was used to measure the DOP and PER, which were 94.5 % and 40.24 dB, respectively, as seen in Fig. 9.

## 4 Discussion

The findings of this present study clearly indicate that, although the bandgap of ReS_2_ is ∼1.4 eV; which indicates an ∼800 nm operational wavelength; an ReS_2_ SA can still be used to successfully Q-switch fiber lasers at a 1550 nm wavelength [35]. This was largely due to the formation of a sub-bandgap as a result of the inevitable defects acquired during the process of preparing the ReS_2_ SA [36]. The linear absorption spectrum of ReS_2_, as shown in Fig. 3b, still has absorption at 1600 nm, which is far from the effective absorption region of the material. This is because the ReS_2_ possesses strong third order optical nonlinearities leading to a weak linear absorption tail that could extend to longer wavelengths [37]. The findings of this present study were compared to that of previous studies that used ReS_2_ SA to Q-switch fiber lasers. As shown in Table 1, the performance of the proposed laser was comparable to that of existing lasers having a pulse duration as short as 2.8 μs. To the best of our knowledge, this present study is the first to use an ReS_2_ SA as well as all-PM fibers and components to Q-switch a laser. The PM structure of the cavity eliminated the need for a polarization controller and could self-start the Q-switching operation once it reached the threshold power of the pump. Furthermore, as the proposed laser requires a threshold power of only 23 mW to self-start producing Q-switched pulses, it significantly decreases the operating cost in terms of the required voltage and current. The lesser threshold also decreases the intensity of the spontaneous radiation noise produced, thereby, enabling the laser to operate more stably. To date, most studies have only examined the use of ReS_2_-polymer composites as a SA. This deposition method that this present study proposed; i.e., drop-casting the ReS_2_ solution directly onto the fiber connector; it is an easier method of integrating an ReS_2_-based material into the cavity of a laser.

**Table 1:** Q-switched fiber lasers based on ReS_2_ SA.

Gain medium	Deposition technique	ReS_2_ thickness	Nonlinear characterization		Laser parameters							Ref
			$\alpha_{s}$ [%]	$I_{sat}$	λ [nm]	Δλ [nm]	Threshold [mW]	$f_{rep}$ [kHz]	τ [μs]	$P_{avg}$ [mW]	*E* [nJ]	
Yb:fiber	ReS_2_-PDMS film on fiber end	21 nm (30 layers)	44	8.4 MW/ ${\text{cm}}^{2}$	1047	6	56	134	1.56	3.2	13.02	[17]
Er:fiber	ReS_2_ flakes on fiber end	–	–	150 GW/ ${\text{cm}}^{2}$	1532	5	110	64	2.1	2.48	38	[39]
Er:fiber	ReS_2_-PVA film on fiber end	∼ 4 nm (6 layers)	0.12	74 MW/ ${\text{cm}}^{2}$	1557.3	2.4	45	19	5.496	1.2	62,800	[19]
Er:fiber	ReS_2_-PVA film on fiber end	5 nm (7 layers)	–	–	1550	–	120	66.52	2.4	1.25	18.88	[20]
Er:fiber	ReS_2_ solution drop casted on fiber end	∼ 2 nm (3 layers)	1.5	43.9 MW/ ${\text{cm}}^{2}$	1558.4	0.13	23	37.6	2.8	2.2	76.5	This work

The modulation depth of the SA is one of the main parameters that determines the laser performance, and it is affected by the SA material thickness [38]. Lu et al. was able to obtain a high modulation depth of 44 % by stacking 30 layers of ReS_2_ and the shortest pulse duration of 1.56 μs [17]. Although this present study was only able to produce a modulation depth of 1.5 %, it can be enhanced by increasing the thickness of the layered ReS_2_ material.

## 5 Conclusion

In summary, this study proposed a linearly polarized Er-doped fiber laser that was capable of producing Q-switched pulses by inserting a ReS_2_ SA in a ring cavity that was made of all-PM fibers and components. The Q-switched pulses that the proposed laser produced had a center wavelength and 3-dB bandwidth of 1558.4 nm and 0.13 nm, respectively. The minimum duration of the output pulses was 2.8 μs while its energy and repetition rate were 76.5 nJ and 37.6 kHz, respectively. Furthermore, as the cavity of the proposed laser was created solely using all-PM components and fibers, the beam that it produced was linearly polarized and had an average DOP and PER of 94.5 % and 40.24 dB, respectively. Lastly, this present study is, potentially, the first to propose a fiber laser that is capable of producing Q-switched pulses using an ReS_2_ SA and an all-PM cavity.

## Data availability statement

Data will be made available on request.

## CRediT authorship contribution statement

**Mahmoud Muhanad Fadhel:** Writing – original draft. **Abdulwahhab Essa Hamzah:** Writing – review & editing. **Norazreen Abd Aziz:** Writing – review & editing. **Mohd Saiful Dzulkefly Zan:** Writing – review & editing. **Norhana Arsad:** Writing – review & editing, Project administration.

## Declaration of competing interest

The authors declare that they have no known competing financial interests or personal relationships that could have appeared to influence the work reported in this paper.
